# Targeting the NEK7/NLRP3 Inflammasome Axis: Synergistic Protection of Intravitreal MCC950 and Systemic Metformin Against Diabetic Retinopathy in Rats

**DOI:** 10.1002/edm2.70151

**Published:** 2026-01-26

**Authors:** Kexuan Ren, Xiaofeng Li

**Affiliations:** ^1^ Dalian University Affiliated Xinhua Hospital Dalian liaoning China

**Keywords:** apoptosis, diabetic retinopathy, MCC950, metformin, NEK7, NLRP3 inflammasome, oxidative stress, synergistic therapy

## Abstract

**Objective:**

Diabetic retinopathy (DR) is characterised by chronic neuroinflammation where the NLRP3 inflammasome plays a pivotal role. This study investigated the therapeutic potential and underlying mechanism of combining systemic metformin (MET) with intravitreal MCC950, a specific NLRP3 inhibitor, in a rodent model of DR.

**Methods:**

A type 2 diabetic rat model was induced by high‐fat diet and streptozotocin (STZ) injection. Diabetic rats were divided into DR, MET, MCC950 and MET+MCC950 treatment groups. Body weight and blood glucose were monitored. Retinal structural changes were assessed by HE and PAS staining. Apoptosis was detected by TUNEL assay, and oxidative stress was evaluated by ROS fluorescence. The expression and interaction of key proteins within the NEK7/NLRP3 pathway were analysed by Western blot and immunofluorescence.

**Results:**

The DR group exhibited significant retinal thinning, increased acellular capillaries, elevated apoptosis and oxidative stress. While monotherapies showed partial improvement, the MET+MCC950 combination yielded the most robust protective effects, nearly restoring retinal morphology and significantly reducing apoptosis and ROS levels. Mechanistically, combination therapy most effectively suppressed the activation of the NEK7/NLRP3 inflammasome pathway, as evidenced by decreased protein levels of NEK7, NLRP3, ASC, cleaved‐Caspase‐1 and IL‐1β. Immunofluorescence confirmed enhanced NEK7/NLRP3 interaction in DR, which was markedly inhibited by the combination treatment. A significant positive correlation was found between ROS levels and NEK7 expression.

**Conclusion:**

The study demonstrates that the combination of systemic metformin and intravitreal MCC950 confers superior protection against DR by synergistically inhibiting the NEK7/NLRP3 inflammasome pathway, resulting in reduced oxidative stress, apoptosis and inflammatory response. This novel combinational strategy presents a promising therapeutic approach for DR.

## Graphical abstract

Graphical Abstract

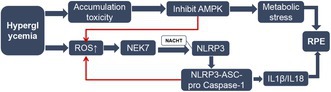

## 1 Introduction

Diabetic retinopathy (DR), one of the most prevalent and devastating microvascular complications of diabetes mellitus, stands as the leading cause of acquired blindness globally, inflicting irreversible vision loss on millions of patients annually and imposing an enormous burden on public health systems and clinical practice worldwide [1, 2]. DR is now recognised as more than a vascular disorder. It is fundamentally a ‘neurovascular disease’, where chronic low‐grade inflammation, oxidative stress and metabolic dysregulation converge. This self‐perpetuating cascade drives concurrent retinal neurodegeneration and vascular injury, ultimately exacerbating disease progression [3, 4, 5]. This updated mechanistic understanding underscores a critical limitation of current therapeutic paradigms: while standard interventions—including intravitreal anti‐vascular endothelial growth factor (VEGF) agents, laser photocoagulation and corticosteroids—effectively alleviate late‐stage pathological manifestations such as macular oedema and neovascularisation, they fail to target the core inflammatory drivers that initiate and sustain retinal damage. Consequently, suboptimal therapeutic responses or disease recurrence are common in a substantial subset of patients [6, 7, 8], highlighting an urgent unmet clinical need for mechanism‐based therapies that address the root causes of DR pathogenesis.

Aberrant activation of innate immune signalling pathways lies at the heart of retinal inflammation in DR, with the nucleotide‐binding domain, leucine‐rich repeat, and pyrin domain‐containing 3 (NLRP3) inflammasome emerging as a pivotal mediator [9, 10]. As a multicomponent cytosolic complex, the NLRP3 inflammasome is potently activated within the proinflammatory microenvironment of DR. Upon activation, it orchestrates the cleavage of caspase‐1, which in turn drives the maturation and secretion of the proinflammatory cytokines interleukin‐1β (IL‐1β) and interleukin‐18 (IL‐18) [11, 12]. These cytokines propagate a cascade of retinal cell damage—including endothelial dysfunction, neuroglial activation and barrier disruption—thereby amplifying pathological progression. Notably, NIMA‐related kinase 7 (NEK7) has recently been identified as an essential upstream regulator of NLRP3 inflammasome assembly and activation: it directly modulates the conformational transition of NLRP3, a step that is indispensable for initiating inflammatory signalling [13]. This discovery not only deepens our comprehension of DR's inflammatory mechanisms but also identifies NEK7/NLRP3 as a promising therapeutic axis. However, intervention strategies targeting this axis—particularly combinatorial approaches that integrate systemic metabolic regulation with local retinal inflammation suppression—remain largely unexplored in the context of DR.

Metformin, the first‐line pharmacotherapeutic for type 2 diabetes, exerts pleiotropic effects that extend well beyond glycemic control. Cumulative evidence demonstrates its robust anti‐inflammatory and antioxidant properties, many of which are mediated by the activation of AMP‐activated protein kinase (AMPK) [14]. Critically, AMPK activation directly inhibits NLRP3 inflammasome activity [15], suggesting that metformin may confer retinal protection in DR through an ‘immunometabolic regulation’ mechanism [16]. Furthermore, its systemic action ameliorates the global metabolic dysregulation inherent to diabetes, making it an ideal candidate for synergistic combination with localised therapies. In contrast, MCC950 is a highly selective small‐molecule inhibitor of the NLRP3 inflammasome that has shown remarkable anti‐inflammatory efficacy in preclinical models of diverse inflammatory disorders, including rheumatoid arthritis and sepsis [17, 18]. Moreover, existing research indicates that the combination of metformin with MCC950 exerts a protective effect in DSS‐induced colitis [19]. The combination therapy demonstrates synergistic effects [20]. Despite this promise, critical gaps persist in its application to DR: the efficacy of intravitreal administration (a route that enables direct targeting of retinal lesions), the optimal therapeutic concentration, and its potential synergism with systemic agents like metformin have not been systematically validated.

Against this backdrop, we propose the central hypothesis: Systemic metformin administration combined with intravitreal MCC950 injection will synergistically attenuate DR progression by concurrently targeting two pivotal pathogenic axes of DR—metabolic dysfunction and focal NLRP3 inflammasome activation. This combinatorial strategy leverages metformin's broad systemic effects on metabolic and inflammatory homeostasis, paired with MCC950's precise, localised inhibition of the NLRP3 inflammasome. In the present study, we utilised a rodent model of DR to (1) validate the retinal protective efficacy of this combination therapy and (2) elucidate its underlying molecular mechanisms, with a specific focus on the NEK7/NLRP3 signalling axis. Our findings aim to provide novel insights into a mechanism‐based combinatorial pharmacologic strategy for DR, laying the experimental groundwork for the development of more effective and sustainable clinical translation approaches.

## 2 Materials and Methods

### 2.1 Animals and Ethical Statement

Thirty six‐week‐old male Sprague–Dawley rats (weight: 220 ± 20 g) were purchased from Liaoning Changsheng Biotechnology Co. Ltd., situated in Liaoning, China (Licence No.: SCXK(Liao)2020–0001). Animals were housed under specific pathogen‐free conditions at a controlled temperature (23.5°C ± 1.5°C) and humidity (50%–70%) with ad libitum access to food and water. All experimental procedures were approved by the Animal Ethics Committee of the Dalian University Affiliated Xinhua Hospital (Approval No.: 2025–017‐01) and conducted in accordance with the ARVO Statement for the Use of Animals in Ophthalmic and Vision Research.

### 2.2 Reagents and Antibodies

MCC950 was obtained from MedChemExpress (MCE). Metformin was purchased from Shijiazhuang Yiling Pharmaceutical Co. Ltd. Forty‐five per cent of high‐fat diet (HFD) was supplied by Shuyu Biotechnology. Streptozotocin (STZ), haematoxylin and eosin (HE) staining kit, periodic acid–Schiff (PAS) staining kit, protease K, TUNEL apoptosis detection kit, protease inhibitor cocktail, phenylmethylsulfonyl fluoride (PMSF), RIPA lysis buffer, BCA protein assay kit, reactive oxygen species (ROS) assay kit and antifade mounting medium were all sourced from Beyotime Biotechnology. Eyeball fixative and PVDF membranes were obtained from Servicebio. SuperSignal West Pico PLUS ECL substrate was from Melun Biotechnology. All primary antibodies against NLRP3, NEK7, ASC, Caspase‐1, Cleaved‐Caspase‐1, IL‐1β and Tubulin were purchased from Affinity Biosciences.

### 2.3 Diabetic Retinopathy Model and Experimental Design

After 2 weeks of acclimatisation, rats were randomly assigned using a random number table to one of five groups (*n* = 6 per group): Normal control (NC), diabetic retinopathy (DR), Metformin (MET, 300 mg/kg/d by gavage), MCC950 (intravitreal injection) and combination (MET + MCC950) groups. Type 2 diabetes was induced in all groups except NC by feeding a 45% high‐fat diet (HFD) for 6 weeks followed by a single intraperitoneal injection of streptozotocin (STZ, 40 mg/kg) after a 16‐h fast. Diabetes was confirmed 72 h post‐STZ by random blood glucose ≥ 16.7 mmol/L, sustained alongside classic symptoms (polyuria, polydipsia and weight loss). DR development was confirmed after 8 weeks of sustained hyperglycemia by the presence of retinal microaneurysms observed under microscopy and subsequent quantitative analysis of acellular capillaries via PAS staining.

### 2.4 Drug Administration and Sample Collection

From week 16, the MET and MET+MCC950 groups received daily metformin (300 mg/kg, p.o.), while other groups received an equal volume of vehicle. The MCC950 and MET+MCC950 groups received weekly intravitreal injections of MCC950 (2 μL/eye) for 4 weeks, which was dissolved in DMSO and further diluted in PBS [21] to a final concentration of 1 mM. NC and DR groups received intravitreal injections of vehicle. To minimise bias, the intravitreal injections were performed by an investigator blinded to the group allocations.

After the 4‐week treatment period, rats were euthanised under deep anaesthesia. For each animal, one eye was immediately fixed in paraformaldehyde for subsequent histological analysis (HE, IHC, IF and PAS staining), and the contralateral eye was dissected to harvest and snap‐freeze the retina for Western blot analysis. All subsequent quantitative assessments, including histological evaluation, image analysis and molecular data processing, were conducted by investigators blinded to the experimental groups.

### 2.5 Metabolic Monitoring and Histological Assessment

Body weight and random blood glucose levels were monitored every 2 weeks. Retinal morphology was assessed by HE staining, and vascular pathology was evaluated by PAS staining of trypsin‐digested retinal vascular networks according to standard protocols.

### 2.6 Immunohistochemistry (IHC), TUNEL and ROS Staining

IHC for ASC was performed on paraffin sections using a standard protocol with antigen retrieval and DAB development. Mean optical density was measured using ImageJ software on five non‐overlapping fields from the ganglion cell layer and inner nuclear layer per section. Three sections per retina and six retinas per group were analysed. Apoptosis was detected using a TUNEL assay kit according to the manufacturer's instructions. The apoptosis index was calculated as the percentage of TUNEL‐positive cells relative to total nuclei in five randomly selected fields per retina. Intracellular ROS levels were assessed using dihydroethidium (DHE) staining on frozen retinal sections. Sections were incubated with 5 μM DHE in a dark, humidified chamber at 37°C for 30 min, then washed with PBS and immediately imaged under a fluorescence microscope.

### 2.7 Western Blot Analysis

Retinal tissues were homogenised in RIPA buffer containing protease inhibitors. Protein concentrations were determined by BCA assay. Equal amounts of protein were separated by SDS‐PAGE, transferred to PVDF membranes and probed with specific primary antibodies overnight at 4°C, followed by incubation with HRP‐conjugated secondary antibodies. Protein bands were visualised using ECL reagent and quantified by densitometry using ImageJ software.

### 2.8 Immunofluorescence (IF)

For IF double‐labelling, retinal sections were incubated with primary antibodies against NEK7/NLRP3 or NLRP3/IL‐1β overnight at 4°C, followed by incubation with appropriate fluorescent secondary antibodies. Nuclei were counterstained with DAPI. Images were captured using a fluorescence microscope and analysed with ImageJ.

### 2.9 Statistical Analysis

Data are presented as mean ± SEM. Statistical analyses were performed using IBM SPSS Statistics 27.0 and GraphPad Prism 10.0. Body weight and blood glucose data were analysed by repeated measures ANOVA. Other data were compared by one‐way ANOVA followed by LSD post hoc test for multiple comparisons. The correlation between two variables was assessed across all animals in the study. Pearson's correlation coefficient (r) was calculated to determine the strength and direction of the linear relationship. A value of *p* < 0.05 was considered statistically significant.

## 3 Results

### 3.1 Successful Establishment of a Type 2 Diabetic Retinopathy Model in SD Rats Induced by STZ

**FIGURE 1:**
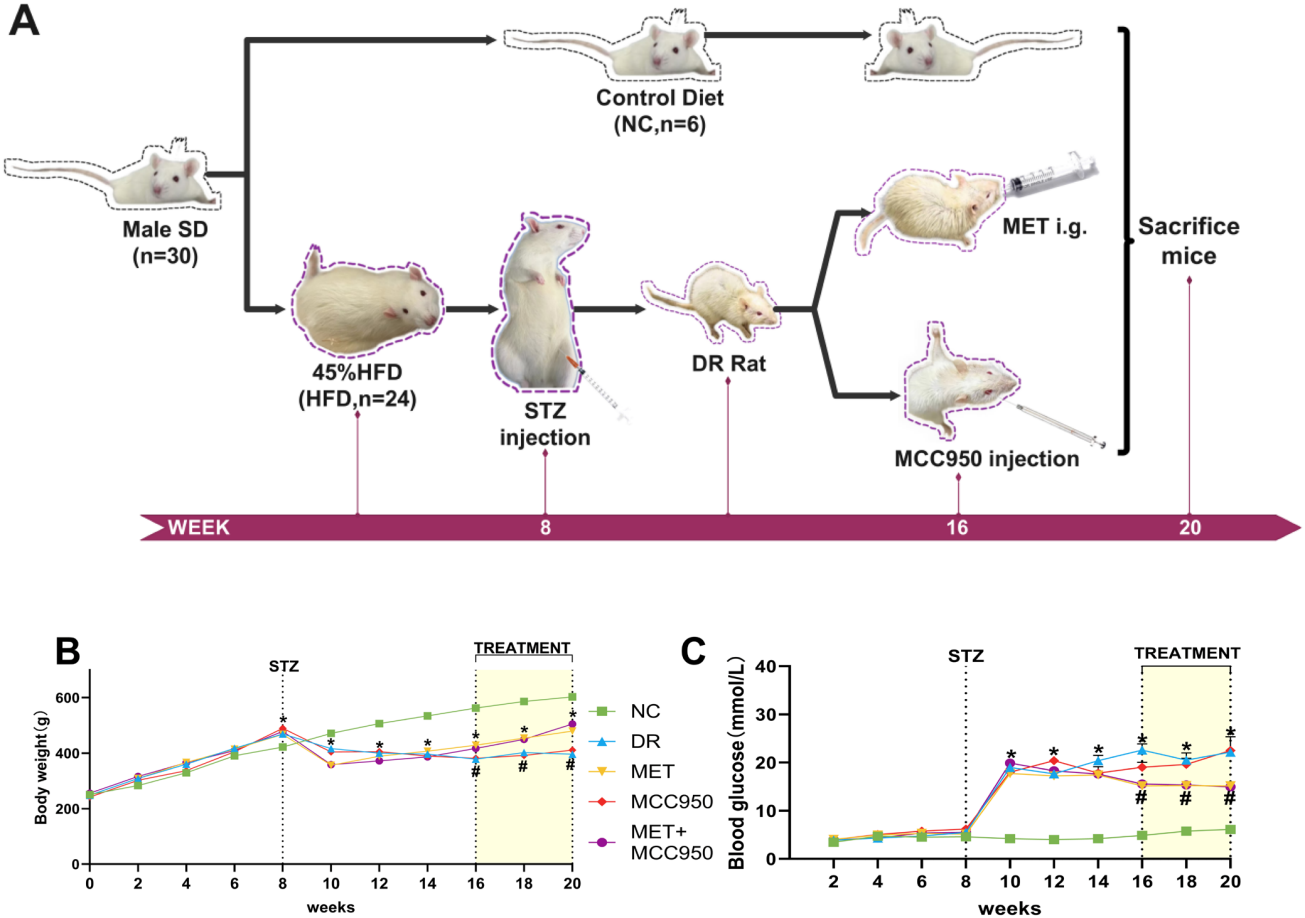
Body weight and blood glucose changes in STZ‐induced type 2 diabetic SD rats. (A) Experimental Flowchart and Timeline. (B) Body weights of rats in different groups. (C) Blood glucose of rats in different groups. Data are shown as mean ± SEM, *n* = 6 per group. Compared with NC group: *p < 0.05; NC: Normal Control group. DR: Diabetic Retinopathy group; MET: Diabetic retinopathy group treated with Metformin; MCC950: Diabetic retinopathy group treated with MCC950; MET+MCC950: Diabetic retinopathy group treated with Metformin and MCC950.

**FIGURE 2:**
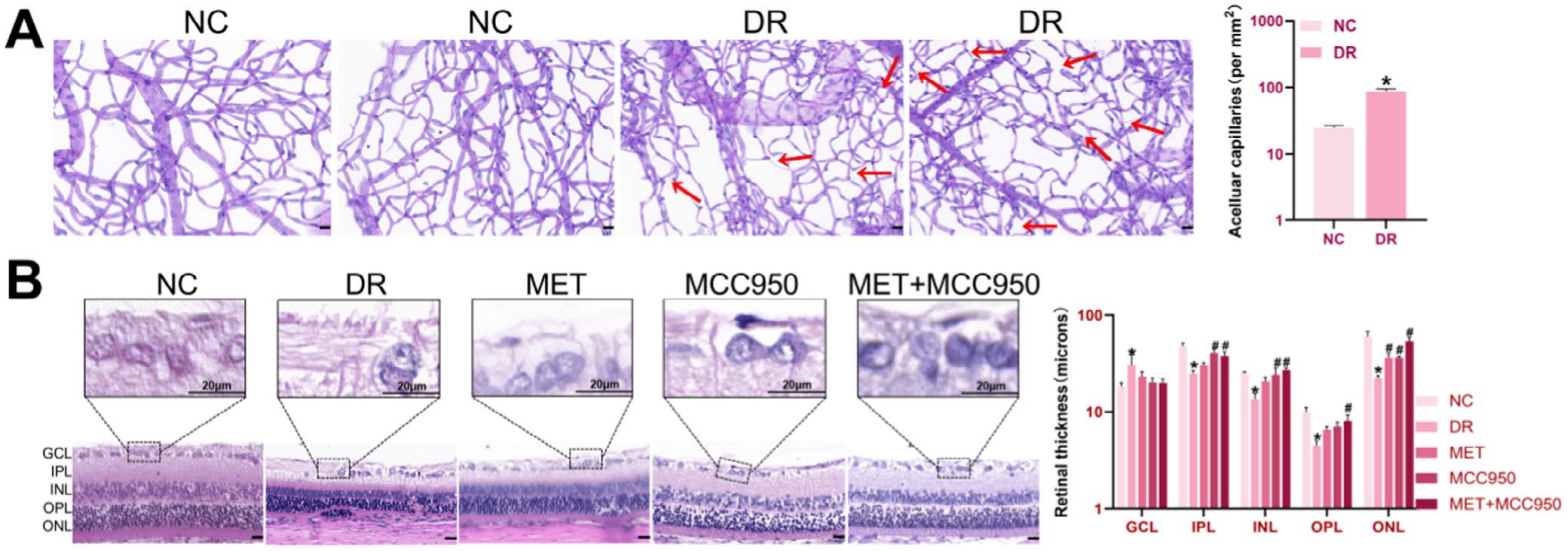
Morphological Changes in Retinal Tissue Among Different Groups of Rats. (A) Representative PAS‐stained retinal whole‐mounts and quantitative analysis of acellular capillaries. (B) HE staining for structural changes in the rat retina and analysis of retinal layer thickness across groups. Data are shown as mean ± SEM, *n* = 6 per group. Compared with NC group: *p < 0.05; NC: Normal Control group. DR: Diabetic Retinopathy group; MET: Diabetic retinopathy group treated with Metformin; MCC950: Diabetic retinopathy group treated with MCC950; MET+MCC950: Diabetic retinopathy group treated with Metformin and MCC950. GCL: Ganglion cell layer; IPL: Inner plexiform layer; INL: Inner nuclear layer; OPL: Outer plexiform layer; ONL: Outer nuclear layer. Scale bars for all images are 20 μm.

A type 2 diabetic rat model was established using 6–8‐week‐old male SD rats fed a 45% high‐fat diet followed by intraperitoneal injection of STZ. The experimental timeline and detailed procedures are illustrated in Figure 1A. Compared to the NC group, rats in the other groups exhibited significant weight loss and sustained hyperglycemia (blood glucose ≥ 16.7 mmol/L) after STZ injection (all *p* < 0.05; Figure 1B,C). Increased water intake, food consumption and urine output were also observed, indicating successful induction of type 2 diabetes. After 8 weeks of continued high‐fat feeding, rats with observable fundus lesions were selected, and retinal tissues from these diabetic rats and NC rats were subjected to PAS staining. The results showed a significant increase in acellular capillaries in the diabetic rat retinas compared to the NC group (indicated by red arrows, Figure 2A), suggesting pathological retinal neovascularisation and confirming the successful establishment of DR.

### 3.2 MET Combined With MCC950 Treatment Ameliorated Metabolic Parameters in DR Rats

Body weight and blood glucose levels were monitored every 2 weeks throughout the experiment. During weeks 2–8 of high‐fat diet feeding, the DR, MET, MCC950 and MET + MCC950 groups showed increased body weight compared to the NC group. After STZ injection (weeks 10–16), body weight in these groups decreased significantly relative to the NC group (*p* < 0.05). Starting from week 16, pharmacological interventions were administered. Compared to the NC group, body weight remained significantly lower in the DR and MCC950 groups (*p* < 0.05), whereas the MET and MET + MCC950 groups showed significant recovery in body weight compared to the DR group (*p* < 0.05; Figure 1B).

No significant differences in blood glucose were observed among the groups during weeks 2–8. After STZ injection (weeks 10–16), blood glucose levels were significantly elevated in the DR, MET, MCC950 and MET + MCC950 groups compared to the NC group (all *p* < 0.05). After treatment initiation, blood glucose remained high in the DR and MCC950 groups compared to NC (*p* > 0.05), but was significantly reduced in the MET and MET + MCC950 groups compared to the DR group (*p* < 0.05; Figure 1C).

### 3.3 MET Combined With MCC950 Treatment Attenuated Retinal Structural Damage in DR Rats

HE staining revealed clear retinal layers and well‐organised cellular arrangement in the ganglion cell layer (GCL), inner nuclear layer (INL) and outer nuclear layer (ONL) in the NC group (Figure 2B). In contrast, the DR group showed disorganised retinal architecture, GCL oedema with reduced cell count, significant thinning of remaining layers (*p* < 0.05), disordered cell arrangement and cytoplasmic hypereosinophilia.

All treatment groups showed varying degrees of improvement: the MET group exhibited alleviated GCL oedema, increased INL and ONL thickness, and more regular cell arrangement; the MCC950 group displayed clearer retinal lamination, significantly reduced GCL oedema, visible perinuclear halos, and markedly increased INL and ONL thickness; the MET + MCC950 group showed the most substantial recovery, with clear retinal layers, regular cell alignment and near‐normal thickness (*p* < 0.05; Figure 2C).

### 3.4 Immunohistochemical Detection of ASC Expression

Immunohistochemical analysis revealed significant differences in the average optical density of ASC among the groups (all *p* < 0.05). Strong ASC‐positive expression (brown‐yellow staining) was observed throughout retinal layers in the DR group compared to NC. All treatment groups showed significantly reduced ASC expression compared to the DR group, with the most pronounced reduction in the MET + MCC950 group: the MET group showed overall reduced expression, the MCC950 group exhibited positive signals mainly in the INL and ONL, and the MET + MCC950 group showed only faint positive staining (all *p* < 0.05; Figure 2D,E).

### 3.5 MET Combined With MCC950 Treatment Suppressed Retinal Apoptosis in DR Rats

**FIGURE 3:**
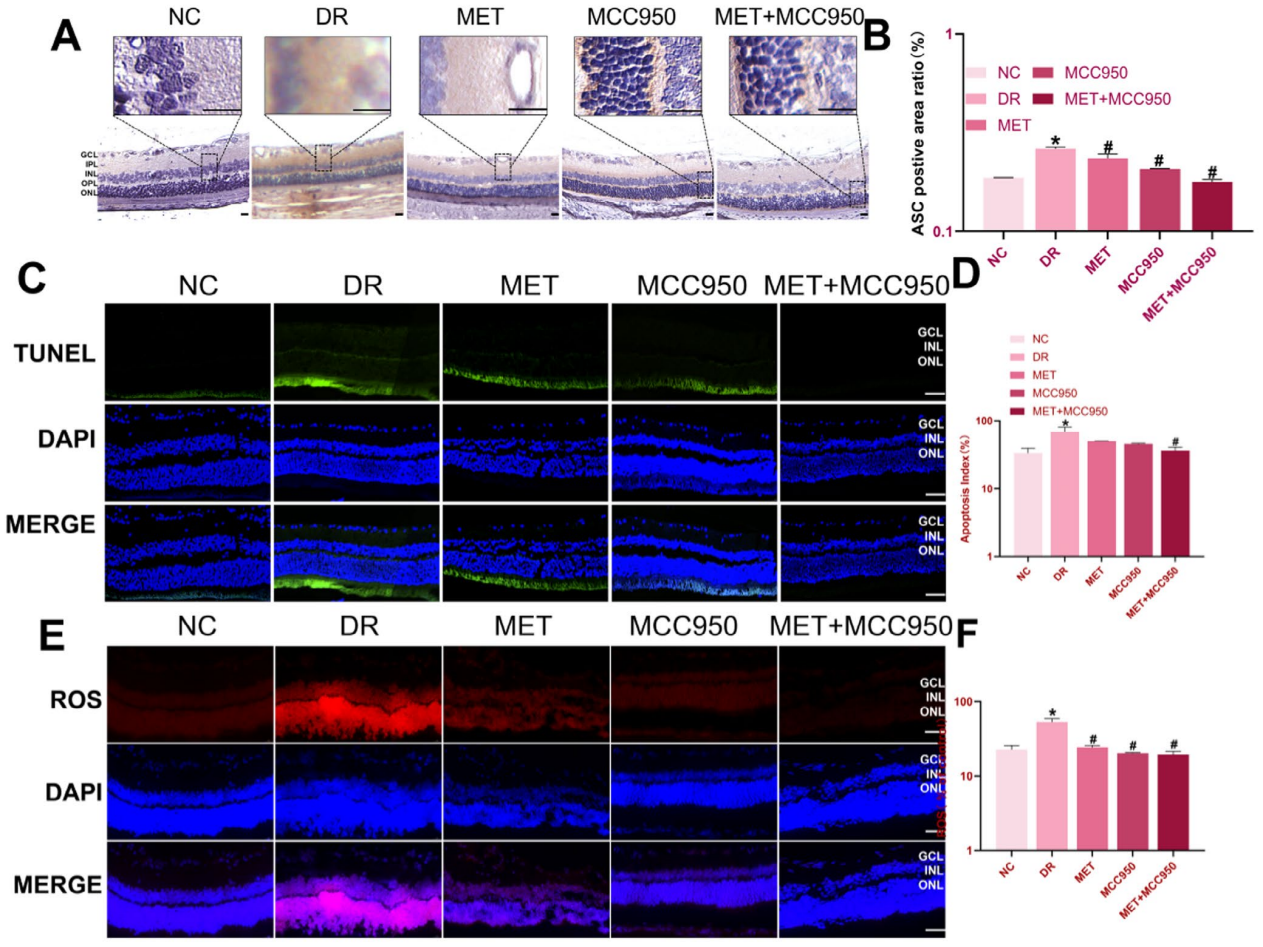
MET Combined with MCC950 Inhibits Apoptosis and Oxidative Stress in Retinal Cells of DR Rats. (A–B) Immunohistochemical staining and results analysis of rat retinas. (C–D) TUNEL assay for detecting apoptosis in retinal cells and quantitative analysis across groups. (E–F) Detection and quantitative analysis of ROS levels in retinal tissues across groups. Data are shown as mean ± SEM, *n* = 6 per group. Compared with NC group: *p < 0.05; NC: Normal Control group. DR: Diabetic Retinopathy group; MET: Diabetic retinopathy group treated with Metformin; MCC950: Diabetic retinopathy group treated with MCC950; MET+MCC950: Diabetic retinopathy group treated with Metformin and MCC950. Scale bars for all images are 50 μm.

TUNEL staining was used to detect apoptotic cells (green fluorescent nuclei). The NC group showed only sporadic TUNEL‐positive cells, while the DR group exhibited a significantly higher density and intensity of TUNEL staining (*p* < 0.05; Figure 3). Both MET and MCC950 monotherapies significantly reduced the percentage of apoptotic cells, with the most potent anti‐apoptotic effect observed in the MET + MCC950 combination group (*p* < 0.05; Figure 3), indicating that MCC950 exerts anti‐apoptotic effects alongside its role in inhibiting NLRP3 inflammasome activation.

### 3.6 MET Combined With MCC950 Treatment Reduced Oxidative Stress in the Retinas of DR Rats

**FIGURE 4:**
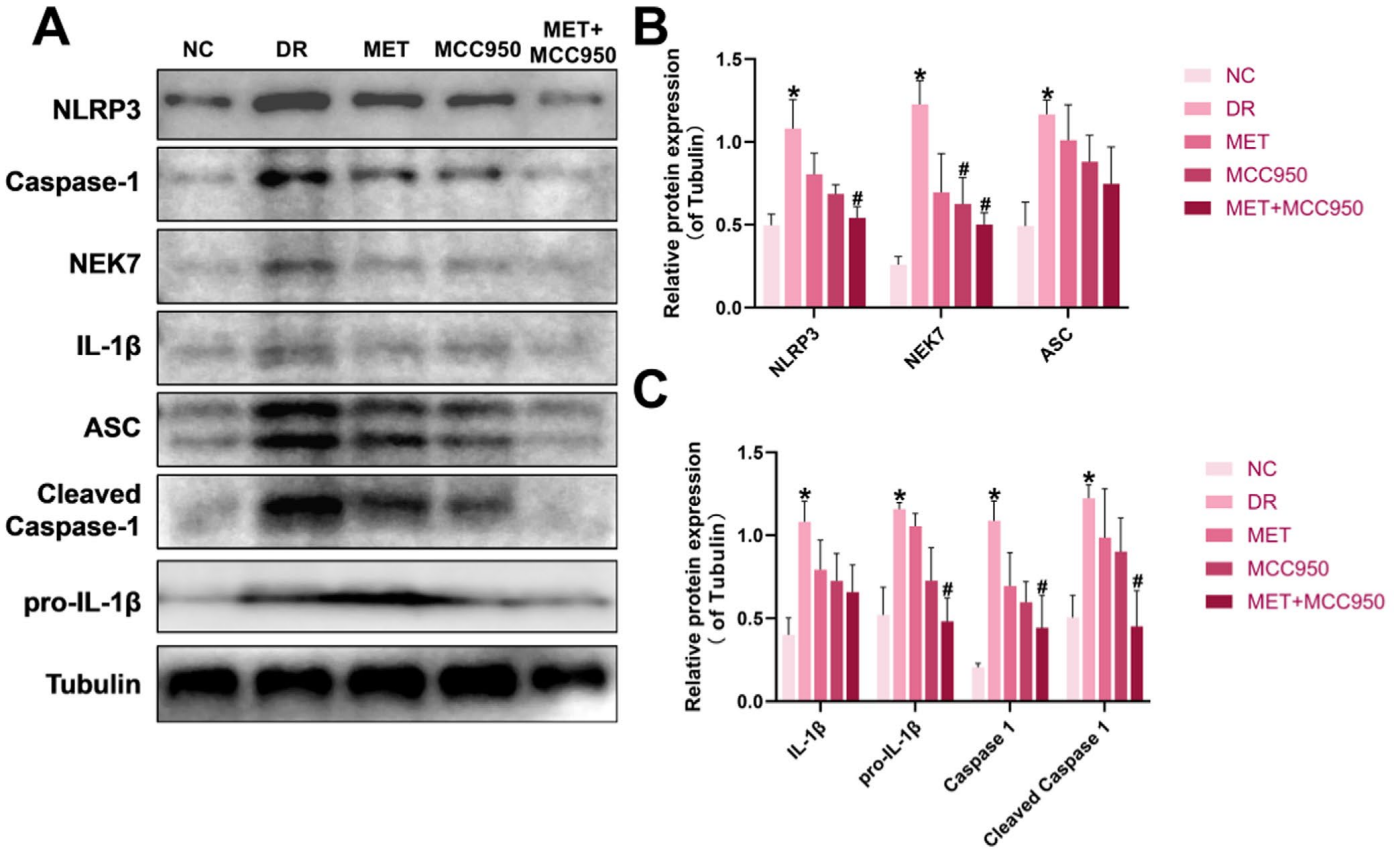
MET combined with MCC950 reduces NLRP3 inflammasome protein expression in retinal tissue of DR rats via the NEK7/NLRP3 pathway. (A) Retinal NLRP3 inflammasome protein expression in each group of rats. (B–C) Analysis of retinal NLRP3 inflammasome protein expression results in each group of rats. Data are shown as mean ± SEM, *n* = 6 per group. Compared with NC group: *p < 0.05; NC: Normal Control group; DR: Diabetic Retinopathy group; MET: Diabetic retinopathy group treated with Metformin; MCC950: Diabetic retinopathy group treated with MCC950; MET+MCC950: Diabetic retinopathy group treated with Metformin and MCC950.

ROS levels (red fluorescence) were low in the NC group but significantly enhanced in the DR group, particularly distributed in the INL and ONL (*p* < 0.05; Figure 4), indicating elevated oxidative stress under diabetic conditions. Both MET and MCC950 reduced ROS levels, with the most effective reduction achieved by the MET + MCC950 combination (*p* < 0.05), demonstrating that co‐treatment effectively ameliorates oxidative damage in DR.

### 3.7 MET Combined With MCC950 Treatment Inhibited NLRP3 Inflammasome Activation in Diabetic Retinas

Western blot analysis showed that protein expression levels of NLRP3, NEK7, ASC, Caspase‐1 and IL‐1β were significantly elevated in the DR group compared to NC (*p* < 0.05). MET or MCC950 monotherapy downregulated the expression of these proteins, with the most significant suppression observed in the MET + MCC950 combination group (*p* < 0.05; Figure 4), indicating that co‐treatment effectively inhibits NLRP3 inflammasome activation and pyroptosis.

### 3.8 MET Combined With MCC950 Treatment Inhibits NLRP3 Inflammasome Expression via the NEK7/NLRP3 Pathway

**FIGURE 5:**
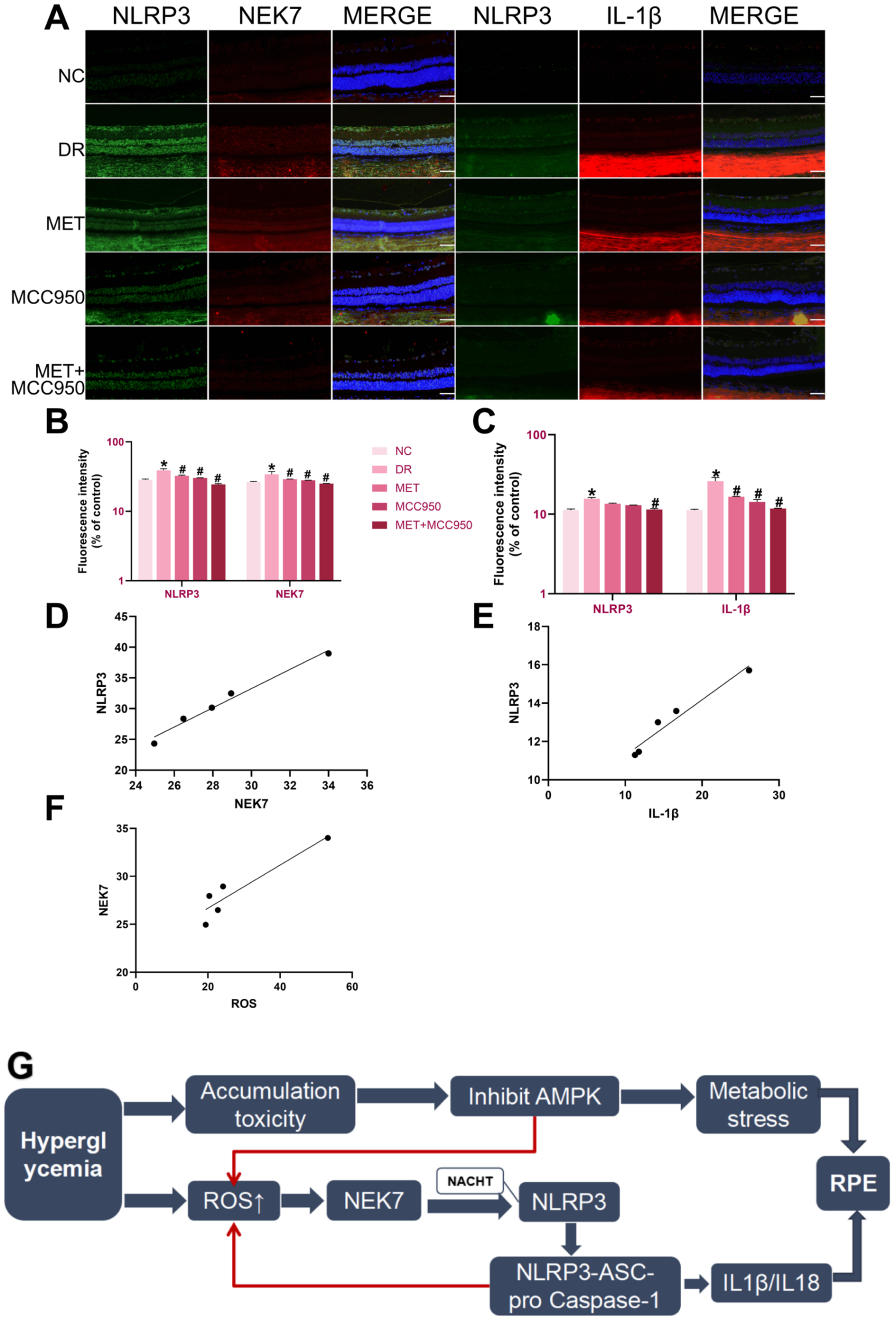
Effects of MET Combined with MCC950 on the NEK7/NLRP3 Pathway in Retinal Tissue of DR Rats. (A) Double staining of NLRP3 and NEK7, and NLRP3 and IL‐1β in rat retinas using immunofluorescence. (B) Analysis of fluorescence intensity expression of NLRP3 and NEK7 double staining in rat retinas. (C) Analysis of fluorescence intensity expression for NLRP3 and IL‐1β double staining in rat retinas. (D) Correlation analysis of NLRP3 and NEK7 fluorescence expression in rat retinas. (E) Correlation analysis of NLRP3 and IL‐1β fluorescence expression in rat retinas. (F) Correlation analysis of ROS and NEK7 fluorescence expression in rat retinas. (G) Schematic diagram of hyperglycemia‐induced NLRP3 inflammasome activation via the ROS/NEK7 pathway. Data are shown as mean ± SEM, *n* = 6 per group. Compared with NC group: *p < 0.05; NC: Normal Control group. DR: Diabetic Retinopathy group; MET: Diabetic retinopathy group treated with Metformin; MCC950: Diabetic retinopathy group treated with MCC950; MET+MCC950: Diabetic retinopathy group treated with Metformin and MCC950. Scale bars for all images are 50 μm.

Immunofluorescence double labelling showed significantly enhanced co‐localisation of NLRP3 with NEK7 and NLRP3 with IL‐1β in the DR group compared to NC (*p* < 0.05; Figure 5). Drug treatments reduced fluorescence intensity, with the most pronounced effect in the MET + MCC950 group (*p* < 0.05), confirming that the combination therapy effectively suppresses high glucose‐induced NEK7 expression and NLRP3 inflammasome activation through the NEK7/NLRP3 pathway.

Pearson correlation analysis revealed a significant positive correlation between NEK7 and NLRP3 levels (*r* = 0.9785, *p* < 0.01), as well as between IL‐1β and NLRP3 levels (*r* = 0.9477, *p* < 0.01), supporting the critical roles of NEK7 and IL‐1β in NLRP3 inflammasome activation. Additionally, a significant positive correlation was observed between ROS levels and NEK7 immunofluorescence intensity (*r* = 0.8659, *p* < 0.05; Figure 5), suggesting that oxidative stress may contribute to NEK7 upregulation in the diabetic retina.

## 4 Discussion

This study demonstrates that the combination of systemic metformin and intravitreal MCC950 provides superior protection against DR by synergistically inhibiting the NEK7/NLRP3 inflammasome pathway. Our findings reveal that this combinatorial approach not only ameliorates metabolic dysfunction but also potently alleviates retinal inflammation, oxidative stress and apoptotic cell death, achieving significantly enhanced efficacy compared to monotherapies.

A key innovation of our study lies in proposing a ‘prime and inhibit’ therapeutic strategy. Metformin, beyond its established glycemic benefits, may prime the retinal microenvironment through AMPK activation—a mechanism known to indirectly suppress NLRP3 priming and mitigate systemic inflammation [16, 19, 20]. MCC950, in turn, acts as a highly specific NLRP3 inhibitor, preventing inflammasome assembly. The observed synergy underscores the importance of concurrently targeting both systemic metabolic disturbances and localised inflammatory responses to disrupt the self‐perpetuating damage cycle in DR.

Mechanistically, our data highlight NEK7 as a critical upstream regulator in diabetic retinopathy. Under diabetic conditions, NEK7 expression was significantly upregulated and strongly correlated with NLRP3 activation [22, 23]. We propose that hyperglycemia‐induced oxidative stress (ROS) triggers NEK7 upregulation, establishing a feed‐forward loop that amplifies inflammasome signalling—a notion supported by the significant positive correlation between ROS and NEK7 levels [24]. The combination therapy most effectively disrupted this NEK7‐NLRP3 interaction, providing a novel mechanistic basis for its superior efficacy. This is consistent with the findings of Zhang's team [25].

This synergistic suppression translated into comprehensive functional benefits, including the most substantial reductions in pro‐inflammatory cytokines, oxidative stress markers and apoptotic cell death, along with near‐complete restoration of retinal morphology.

From a translational perspective, our approach holds considerable clinical promise. Repurposing metformin—a safe and widely prescribed medication—for DR prevention represents a pragmatic therapeutic strategy. When combined with intravitreal MCC950 delivery via an established clinical route, this ‘systemic‐local’ combination therapy maximises local drug exposure while minimising systemic side effects, offering a viable paradigm for patients with inadequate responses to current therapies.

### 4.1 Limitations and Future Perspectives

While our data provide compelling correlative evidence for NEK7/NLRP3 axis involvement in the observed synergy, we explicitly state that direct demonstration of NEK7‐NLRP3 physical interaction in our model was not established. Instead, our conclusion is inferential, based on concomitant upregulation, co‐localisation and correlation analyses. Technical constraints precluded co‐immunoprecipitation validation.

Nevertheless, our interpretation is strongly supported by foundational research. Structural studies have definitively established NEK7‐NLRP3 interaction as critical for inflammasome activation [13, 25, 26], and recent work shows that disrupting this interaction effectively suppresses NLRP3 activation [26].

Additional limitations include the absence of electroretinography to assess retinal function. Future studies should incorporate functional assessments and further investigate the molecular crosstalk between AMPK activation and NEK7/NLRP3 inhibition to fully elucidate the therapeutic potential of this pathway in DR.

## 5 Conclusion

In conclusion, our findings establish that co‐treatment with metformin and MCC950 synergistically alleviates diabetic retinopathy by targeting the NEK7/NLRP3 inflammasome pathway through complementary mechanisms. This strategy effectively addresses both the systemic metabolic dysregulation and the local inflammatory drivers of the disease, resulting in robust suppression of oxidative stress, inflammation and apoptosis, and ultimately leading to significant preservation of retinal structure and function. The ‘prime and inhibit’ approach offers a novel, mechanistically grounded, and clinically viable strategy, paving the way for a new class of combinatory therapies for diabetic retinopathy.

## Author Contributions

**Kexuan Ren:** investigation, methodology, formal analysis, data curation, visualization, Writing – original draft. **Li Xiaofeng:** conceptualization, funding acquisition, project administration, supervision, writing – review and editing, and served as the corresponding author.

## Funding

This work was supported by Chinese Medicine Education Association Project, 2024KTM031.

## Ethics Statement

The study was approved by the Scientific Investigation Board of Dalian University Affiliated Xinhua Hospital and was performed in accordance with relevant guidelines and regulations. Laboratory Animal Ethical Review Number: 2025–017‐01.

## Conflicts of Interest

The authors declare no conflicts of interest.

## Supporting information


**Data S1:** Table 1 Contains information regarding the catalogue number of all reagents, and kits used.

## Data Availability

The data that support the findings of this study are available from the corresponding author upon reasonable request.
