# Supplementary material for: Targeting the NEK7/NLRP3 Inflammasome Axis: Synergistic Protection of Intravitreal MCC950 and Systemic Metformin Against Diabetic Retinopathy in Rats
Source: Endocrinol Diabetes Metab. 2026 Jan 26;9(1):e70151. doi: 10.1002/edm2.70151 (PMC12835612; doi:10.1002/edm2.70151)
Supplement: Supplementary file 1 — Data S1: Table 1 Contains information regarding the catalogue number of all reagents, and kits used. [file EDM2-9-e70151-s001.docx]

**Table 1**

Contains information regarding the catalogue number of all reagents,and kits used.

| **Categories** | **vendor** | **catalog number** | **dilution ratio** |
| --- | --- | --- | --- |
| Primary antibodies  NLRP3 | Affinity | BF8029 | 1:1000 (WB)  1:100 (IF) |
| NEK7 | Affinity | DF4467 | 1:1000 (WB)  1:100 (IHC、IF) |
| ASC | Affinity | DF6304 | 1:1000 (WB) |
| Caspase-1 | Affinity | AF5418 | 1:1000 (WB) |
| Cleaved-caspase-1 | Affinity | AF4005 | 1:1000 (WB) |
| IL-1beta | Affinity | AF4006 | 1:1000 (WB)  1:150 (IF) |
| Tubulin | Affinity | AF7011 | 1:1000 (WB) |
| Secondary antibodies  goat anti-mouse IgG | Abcolonal | AS055 | 1:5000 (WB) |
| goat anti-rabbit IgG | Abcolonal | AS056 | 1:5000 (WB) |
| **Reagents** | | **vendor** | **Catalog number** |
| Proteinase K | | Beyotime | ST535 |
| Colorimetric TUNEL Apoptosis Assay Kit | | Beyotime | C1091 |
| BCA protein assay kit | | Beyotime | P0010 |
| ROS assay kit | | Sigma | D7008 |
| Reactive oxygen species assay kit | | Beyotime | S0033S |
| RIPA Lysis Buffer | | Beyotime | P0013B |
| Streptozotocin | | Solarbio | S8050 |
| Hematoxylin-Eosin kit | | Solarbio | G1120 |
| MCC950 | | MCE | HY-12815A |
| Phenylmethanesulfonyl fluoride | | Beyotime | ST505 |
| PVDF membrane | | Millipore | ISEQ00010 |
| FAS eye fixative solution | | Servicebio | G1109 |
| Phosphatase inhibitor cocktail | | Beyotime | P1082 |
| Antifade mounting medium | | Beyotime | P0131 |
| FGSuper Sensitive ECL Luminescence Reagent | | Meilunbio | MA0186 |
| Glycogen Periodic Acid-Schiff Staining Kit | | Servicebio | G1008 |
